# Transporters, an important but poorly studied area of *Toxoplasma gondii*

**DOI:** 10.1186/s13071-025-07216-w

**Published:** 2026-01-25

**Authors:** Liya Wang, Yujuan Jing, Jichao Yang, Xuke Yang, Jiahui Qian, Rui Fang, Fuchun Jian, Longxian Zhang, Senyang Li

**Affiliations:** 1 College of Veterinary Medicine, Henan Agricultural University, No. 218 Longzihu University Area, Zhengdong New District, Zhengzhou, 450046 China; 2 College of Life Sciences, Longyan University, Longyan, Fujian China; 3 Research Center for Infectious Diseases, Department of Pathogen Biology, School of Basic Medical Sciences, Anhui Medical University, Hefei, China; 4 State Key Laboratory of Agricultural Microbiology, College of Veterinary Medicine, Huazhong Agricultural University, Wuhan, Hubei China

**Keywords:** *Toxoplasma gondii*, Host, Transporters, Substrate

## Abstract

Membrane transporters play a vital role in the obligate intracellular parasite *Toxoplasma gondii*, mediating the acquisition of nutrients from host cells, the regulation of ion gradients, and the maintenance of metabolic homeostasis. Despite their central importance for parasite survival, pathogenesis, and drug resistance, the majority of *T. gondii* transporters remain poorly characterized. Key unresolved questions include the mechanisms underlying purine nucleotide transport across the plasma membrane and the import/export of metabolites for core pathways in the apicoplast (e.g., thiamine, isopentenyl diphosphate[IPP]/dimethylallyl diphosphate [DMAPP], and coproporphyrinogen III) and mitochondria (e.g., amino acids and cofactors). Recent advances in bioinformatics and CRISPR-based phenotypic screening have enabled systematic identification of transporter candidates. This review summarizes current knowledge of *T. gondii* transporters localized to the plasma membrane, apicoplast, mitochondria, endoplasmic reticulum, and Golgi apparatus, highlighting their roles in nutrient acquisition, metabolic crosstalk, and organellar function. Furthermore, we propose a screening strategy integrating transmembrane domain prediction, CRISPR phenotyping, and hyperLOPIT-based protein localization to prioritize uncharacterized transporters for functional study. These insights underscore the potential of transporters as therapeutic targets and provide a roadmap for future research into the physiology of *T. gondii*.

**Graphical abstract:**

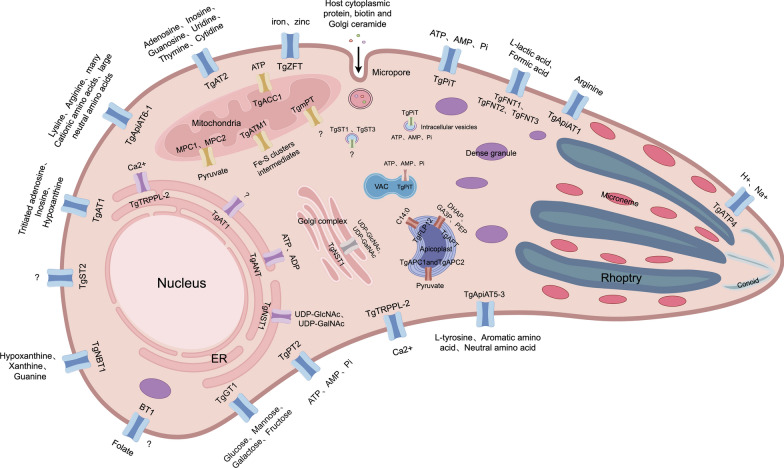

**Supplementary Information:**

The online version contains supplementary material available at 10.1186/s13071-025-07216-w.

## Background

*Toxoplasma gondii* is an obligate intracellular parasite that infects nearly all nucleated mammalian cells, surviving and replicating within a specialized compartment called the parasitophorous vacuole (PV) in the host cytoplasm. Although the PV shields *T. gondii* from host innate immune defenses and lysosomal degradation, it simultaneously restricts access to cytoplasmic nutrients. To acquire essential resources and expel metabolic waste, the parasite must traverse two barriers: the parasitophorous vacuole membrane (PVM) and its own plasma membrane. Unlike the plasma membrane, the PVM is modified by *T. gondii* to form proteinaceous pores (e.g., GRA17, GRA23, and GRA45) [1–3], alongside host-derived structures such as the endosomal sorting complex required for transport (ESCRT) [4, 5] and the host organelle-sequestering tubulostructures (H.O.S.T.) [6]. These adaptations enable passive diffusion of small solutes from the host cytosol into the PV. In contrast, the plasma membrane lacks such permeability and relies on substrate-specific transporters to actively import nutrients (e.g., glucose and amino acids) and export toxic byproducts [7, 8]. These membrane transport proteins are indispensable for parasite survival, as they regulate nutrient uptake, waste extrusion, ion homeostasis, and intercompartmental solute exchange. In addition to the presence of transporters on the plasma membrane of *T. gondii*, numerous transporters are also present in the organelles and are used to maintain the smooth progress of a variety of metabolic pathways. In this review, we systematically searched for relevant articles from the Pubmed (https://pubmed.ncbi.nlm.nih.gov/) and Web of Science databases to summarize current insights into transporters and their substrates, as well as the functions of these substrates in *T. gondii*.

## Plasma membrane transporters: gatekeepers of nutrient acquisition

### Carbohydrate transport

Hexasaccharides, including glucose, are central to cellular metabolism and are important for parasite replication; they maintain energy supply, macromolecular synthesis, and cell surface glycoconjugate production by the uptake of host sugars, all of which are essential for their survival and pathogenicity. In apicomplexan parasites, glucose metabolism is the main source of carbon metabolism and energy for *T. gondii* tachyzoites [8–10]. The glucose transporter *Tg*GT1 is a major, well-characterized hexose transporter located on the plasma membrane of *T. gondii*. In addition to its primary substrate glucose, *Tg*GT1 is also capable of transporting other hexoses such as mannose, galactose, and fructose. Furthermore, tachyzoites express three additional putative sugar transporters (*Tg*ST1–3); however, their specific substrates remain unidentified to date [9]. However, *T. gondii* is highly flexible in carbon metabolism, and loss of *Tg*GT1 causes only a modest growth defect in *T. gondii*, which is able to use host glutamine to provide its carbon skeleton as well as sufficient energy [11–13]. Although glucose and glutamine are the primary carbon sources for *T. gondii*, the parasite can also utilize alternative nutrients such as lactate and alanine in their absence. Critically, all these carbon sources must first be converted to pyruvate through enzymatic reactions to be metabolized further. Therefore, maintaining a continuous pyruvate supply is essential for the survival and metabolism of *T. gondii* [14].

### Phosphate transport

Inorganic phosphate (Pi) is an important nutrient that is essential for the function and regulation of cells, is indispensable for key cellular components of biosynthesis, and is involved in many metabolic and signaling pathways [15, 16]. Inorganic phosphate must be obtained from the host when the parasite infects the host [16]. Three possible phosphate transporters have been identified in *T. gondii*, with *Tg*PiT (P_i_ transporter) and *Tg*PT2 (phosphate transporter 2) located primarily on the plasma membrane and *Tg*mPT (mitochondria phosphate transporter) located in the mitochondria. *Tg*PiT was the first identified high-affinity Na^+^–Pi cotransporter, expressed by the parasite at the plasma membrane for Pi uptake. Further, *Tg*PiT is also localized to inward buds of the endosomal VAC (plant-like vacuole) organelles and some cytoplasmic vesicles. The lack of *Tg*PiT leads to *ΔTgPiT* parasites being impaired in importing Pi and synthesizing polyphosphates. *ΔTgPiT* strains exhibit severe growth defects, such as a reduced cell volume, enlarged VAC organelles, defects in calcium storage, a slightly alkaline pH, and have reduced acute virulence in mice. However, *ΔTgPiT* parasites are still able to form small plaques [17]. Owing to the dual localization of *Tg*PiT on the plasma membrane and the organelles of VAC, and vesicle transport in the cytoplasm, the transporter can reasonably handle the distribution of various ions in *T. gondii*, which would be conducive to maintaining osmotic homeostasis. In recent studies, a novel phosphate transporter, *Tg*PT2, was identified and shown to possess phosphate transport activity, contributing to the parasite’s absorption of inorganic phosphate. The Pi importing activity of *Tg*PT2 could be competitively inhibited by ATP and AMP. Furthermore, direct uptake ability of [^32^P]-ATP was also observed, indicating the parasites’ ability to scavenge host ATP. Although both *Tg*PiT and *Tg*PT2 have phosphate transport activities, only *Tg*PT2 is required for the normal growth of *T. gondii*, whereas *Tg*PiT is dispensable. Whether there is redundancy in transport activity between them is currently unknown. In *Plasmodium*, the expression of PiT alone provides adequate phosphate transport capability [16, 18, 19]. The above results suggest that the transport of phosphate in *T. gondii* and *Plasmodium* may require a variety of different transporters to work together, and that these transporters have a variety of substrate affinities and are able to transport substrates other than phosphate. Moreover, because *Tg*PT2 is restricted to Coccidia parasites and is essential for *T. gondii* survival, it is a potential target for antitoxoplasmic intervention design.

### Amino acid transport

Apicomplexan parasites lack a range of amino acids that must be obtained from host cells through direct uptake or degradation of host proteins [7]. Arginine (Arg) and its downstream metabolites ornithine and polyamines cannot be synthesized de novo and must be obtained from the host [20]. *T. gondii* is unable to synthesize tyrosine (Tyr), tryptophan (Trp), and phenylalanine (Phe) de novo. Although protein degradation provides a minor source of amino acids, the vast majority—including these essential aromatics—must therefore be salvaged from the host via specific transporters [21, 22]. To date, several amino acid transporters critical for *T. gondii* physiology have been characterized. These include *Tg*ApiAT5-3, a tyrosine and aromatic amino acid transporter; *Tg*ApiAT1 (also known as *Tg*NPT1), an arginine transporter; and ApiAT6-1, a lysine transporter. Functional studies have demonstrated that these transporters play vital roles in parasite growth and pathogenicity, both in vitro and in vivo [23–25].*Tg*NPT1, as a highly selective transporter of arginine, transports arginine in the host cytoplasm and is essential for parasite growth and virulence in vitro [26]. The lack of *Tg*NPT1 leads to severe growth impairment of *T. gondii*, but this growth defect is alleviated by the addition of high concentrations of Arg, indicating that there is another arginine transport system in *T. gondii*. Subsequent studies demonstrated the presence of another arginine transporter *Tg*ApiAT6-1 in *T. gondii*.

*Tg*ApiAT6-1 functions as a broad-spectrum cationic amino acid transporter, capable of transporting various substrates including cationic amino acids, large neutral amino acids, and arginine metabolites. Despite this broad activity, it exhibits strong substrate preference. In the presence of lysine, *Tg*ApiAT6-1 preferentially binds and transports this amino acid, while its affinity for other substrates is significantly reduced [20, 27]. Further research has shown that *Tg*ApiAT6-1 and *Tg*ApiAT1 are bidirectional single transporters with the ability to mediate amino acid exchange and promote the accumulation of these two essential cationic amino acids in the cell [20, 27]. *Tg*ApiAT5-3 is a high-affinity transporter of l-tyrosine (L-Tyr). It was found that *Tg*ApiAT5-3 has an unusual dual function, which can not only promote L-Tyr transport into the parasite but also maintain the concentration of aromatic amino acids and macroneutral amino acids in the cell [7, 28].

### Purine salvage and lactate efflux

Purines and pyrimidines are the basic nutrients of all cells, and most eukaryotes can synthesize purines and pyrimidines by themselves. In contrast to pyrimidines, which *T. gondii* is capable of synthesizing de novo, its purine supply exhibits a complete dependence on specific transporters for salvage from the host cell [29–33]. The first identified purine transporter in *T. gondii* was *Tg*AT1 (adenosine transporter, TgGT1_244440), which was shown through the incorporation the radiolabeled purines into the DNA of extracellular *T. gondii* after incubation with tritiated adenosine, inosine, hypoxanthine, and adenine [31, 32]. *Tg*AT1 expressed in vitro showed similar transport activity for inosine, methylmycin B, and allopurinol nucleoside. However, *Tg*AT1 is a low-affinity adenosine transporter (*Km* 120 μM), indicating that other purine transport mechanisms may exist in *T. gondii*. In subsequent studies, other transporters with high-affinity for purine, *Tg*AT2 and *Tg*NBT1, were identified. The substrate of *Tg*NBT1 is hypoxanthine (*Km* 0.91 μM), and is inhibited by guanine and xanthine, which is the first high affinity nucleobase transporter to be identified in an apicomplexan parasite. Another identified nucleoside transporter was *Tg*AT2, which displays a high affinity and could be inhibited by adenosine, inosine, guanosine, uridine, and thymidine (*Km* 0.49 μM) as well as cytidine [30]. However, except for *Tg*AT1, which is annotated in the ToxoDB [34], the proteins of *Tg*AT2 and *Tg*NBT1 have not been found.

A recent phylogenetic re-analysis has led to the redefinition of three equilibrative nucleoside transporters (ENTs) in *T. gondii*—*Tg*ENT1 (TGME49_288540), *Tg*ENT2 (TGME49_500147), and *Tg*ENT3 (TGME49_233130) [35]. Functional characterization revealed that *Tg*ENT2 is bradyzoite-specific and irrelevant for tachyzoite growth, and *Tg*ENT3, although expressed in both life stages, is dispensable. Of these three, *Tg*ENT1 is uniquely essential for parasite survival. A notable finding was that *Tg*ENT1 localizes to the plant-like vacuolar compartment instead of the plasma membrane. In addition, a genetic interaction was observed where co-deletion of *Tg*AT1 and *Tg*ENT3 triggered *Tg*ENT1 overexpression. However, the subcellular localization of *Tg*ENT2 and *Tg*ENT3 remains undetermined, and no functional data exists on the substrate specificity of any of the three *Tg*ENTs. In our recent study, we investigated *Tg*ENT1 (which we named *Tg*NT1) [36]. However, our findings indicate that *Tg*NT1 localizes to the mitochondria, not to the plant-like vacuole. Consistent with the results from the *Tg*ENT1 study, we observed that deletion of *Tg*NT1 leads to parasite lethality and significant alterations in mitochondrial nucleotide levels. Nevertheless, the reason for the distinct subcellular localization observed in the two studies requires further investigation. We propose that the difference in epitope insertion sites may account for the inconsistent localization patterns observed. So, it is unclear whether these newly defined transporters correspond to the previously proposed but unannotated *Tg*AT2 and *Tg*NBT1.

In summary, despite numerous studies on nucleotide transporters in *T. gondii*, a fundamental question remains unresolved: which specific transporter, or combination of transporters, is responsible for the uptake of host-derived purine nucleotides.

### Ion homeostasis

In addition to absorbing nutrients from host cells, *T. gondii* also encodes transporters to transport metabolic waste to host cells. *T. gondii* grows rapidly in the tachyzoite stage, and uses glucose catabolism, mainly through glycolysis, to produce energy for its growth and development [12, 37–39]. The major byproduct of glycolysis, lactic acid, is toxic to the parasite and needs to be extruded into the external medium [40–43]. Members of the microbial formate–nitrite transporter (FNT) family are responsible for monocarboxylate metabolite transport, such as lactate, formate, and its nitrogen analog, nitrite, in bacteria. The FNT family is exclusively present in microorganisms, including prokaryotes and lower eukaryotes, and is not found in mammalian cell membranes. This unique distribution makes FNT a highly attractive and relatively ideal drug target, as inhibitors could achieve selective toxicity against pathogens while minimizing effects on the human host. In apicomplexan parasites, *Pf*FNT was the first identified formate–nitrite transporter that facilitates the efflux of lactate, which, when expressed in *Xenopus laevis* oocytes and yeast, transports both formate and lactate as well as pyruvate and acetate formate [40, 44]. Since *Tg*FNT plays an important role in carbon metabolism in *T. gondii*, the structure of FNT has been deeply studied, and a series of potential inhibitors have been found [45–47]. *T. gondii* possesses three FNT isoforms (*Tg*FNT1–3) that mediate pH-dependent transport of l-lactate and formate across the plasma membrane—a feature distinct from *Plasmodium*. Their expression is stage- and strain-specific: *Tg*FNT1 and *Tg*FNT2 in type I tachyzoites, and *Tg*FNT3 in type II cysts. This differential expression implicates each transporter in supporting specific phases of parasite life cycle progression [42, 48]. In *P. falciparum*, *Pf*FNT is a validated drug target and is essential for parasite proliferation. This stands in sharp contrast to *T. gondii*, where all three *Tg*FNT genes can be disrupted individually or in combination without impairing the lytic cycle under standard in vitro culture conditions [48, 49]. This seems to coincide with the *Tg*FNTs having a higher phenotype score (the phenotype scores for *Tg*FNT1, *TgFNT*2, and *Tg*FNT3 were 1.10, 1.31, and -0.23, respectively) that each of the *Tg*FNTs are dispensable during the lytic cycle [50]. The mechanism by which *T. gondii* tolerates glycolytic lactate production in the absence of all three *Tg*FNT transporters remains unresolved. We hypothesize that this is linked to the parasite’s notable metabolic flexibility. For instance, while glucose and glutamine are the primary carbon sources, *T. gondii* can alternatively utilize lactate and alanine when these are unavailable. Lactate fermentation itself is critical under normal conditions, likely compensating for limited oxidative phosphorylation in the low-oxygen intracellular environment to meet energy demands [14]. Therefore, whether *T. gondii* can metabolize the accumulated lactate and avoid its toxic effects on the parasites still needs further study.

The *Toxoplasma* life cycle includes both intracellular and extracellular phases, and in order to survive in different host environments, they must regulate intracellular sodium ion concentration and pH under widely varying external ion conditions. *Tg*ATP4 is a plasmic sodium pump located on the plasma membrane of *T. gondii* and is important for Na^+^ homeostasis. *Tg*ATP4 is important for the growth of *T. gondii* during multiple lytic cycles, and while not necessary for the parasite to grow, export, or invade inside the cell, it is important for maintaining the viability of the extracellular parasite. *Tg*ATP4 affects the virulence of parasites in the body, and parasites lacking *Tg*ATP4 can still cause disease in the body, but their virulence is reduced. *Tg*ATP4 protein converts H^+^ inward and Na^+^ outward at the same time, and maintains low Na^+^ concentration to maintain Na^+^ homeostasis [51].

Ca^2+^ signals generated by Ca^2+^ entry or intracellular release trigger a cascade of reactions in the parasite that ultimately lead to the occurrence of essential features of the cleavage cycle, such as glide movement, invasion, release, and secretion of proteins required for attachment to host cells [52–54]. Previous studies have shown that Ca^2+^ channel blockers such as nifedipine do not completely inhibit Ca^2+^ influx, and about 20% of Ca^2+^ still enters *T. gondii*, suggesting that there may be more than one Ca^2+^ transport system in the plasma membrane of *T. gondii* [52]. *Tg*TRPPL-2 is the first identified transporter that facilitates calcium uptake into *T. gondii* and Ca^2+^ excretion in ER. It was established that *Tg*TRPPL-2 is important for both plasma membrane Ca^2+^ uptake and ER Ca^2+^ excretion in *T. gondii* tachyzoites. Consistent with previous findings, *Tg*TRPPL-2 deficiency resulted in the impairment of *T. gondii* invasion and egress, leading to the impairment of *T. gondii* growth, which was consistent with the finding that the addition of Ca^2 +^ could promote the egress of *T. gondii* [55]. In addition, *Tg*TRPPL-2 is very sensitive to transient receptor potential (TRP) channel inhibitors and can be inhibited by broad-spectrum TRP channel inhibitors, resulting in impaired growth of *T. gondii* [55]. However, as *Tg*TRPPL-2 is highly conserved with host cell calcium channel proteins, the safety of *Tg*TRPPL-2 as a drug target of *T. gondii* needs to be carefully considered and further studies are warranted.

Metallic cofactors such as iron and zinc are indispensable for the survival and pathogenesis of *T. gondii*, serving as crucial elements in a wide array of enzymes and metabolic pathways [56–59]. However, the mechanisms by which this obligate intracellular parasite acquires these essential metals from the host have long remained enigmatic. Recent breakthroughs have begun to unravel the sophisticated strategies employed by *T. gondii*, involving both the manipulation of host nutrient channels and the function of parasite-encoded specific transporters. *Tg*ZFT (Zn and Fe transporter) was the first identified and characterized iron and zinc importer in *T. gondii*, which localized to the parasite plasma membrane and is essential for the parasite’s life cycle [60]. The function of *Tg*ZFT was validated through functional complementation assays in zinc-transporter-deficient yeast (Δ*zrt1/2* mutant yeast), where its expression rescued the growth phenotype. In contrast, efforts to demonstrate a similar rescue function in iron-transporter-deficient yeast (Δ*fet2/3* mutant yeast) were unsuccessful, failing to provide conclusive evidence for *Tg*ZFT-mediated iron transport. Owing to loss of iron-containing proteins in the mitochondrial electron transport chain, the knockdown of *Tg*ZFT inhibits apicoplast replication and reduces mitochondrial respiration. Although *Tg*ZFT is mainly localized on the plasma membrane, further studies have revealed that *Tg*ZFT localization is dynamic and vacuolar stage dependent. Interestingly, knockdown of *Tg*ZFT triggers partial parasite differentiation, because multiple enlarged, electron-lucent vesicles were observed upon *Tg*ZFT knockdown, which were not present in the untreated cells. It has been previously reported that similar large vesicles contain materials that form the cyst wall, which is a characteristic of *T. gondii* transforming into bradyzoites [61].

### The micropore: a specialized structure for host-derived nutrient uptake

Previous studies have shown that there are invagination structures called micropores in the plasma membrane of *T. gondii* [62–64], and similar microporous structures have also been observed in other apicomplexan parasites such as *Plasmodium* [65] and avian malaria parasites [66]. Micropores of *Plasmodium* have been shown to mediate the uptake of host nutrients [67]. Recent studies have shown that micropore invagination in the plasma membrane is another important channel for *T. gondii* to acquire host nutrients. In addition, a key protein, Kelch13, localized at the dense neck of the organelle, functions as a protein hub at the micropore for endocytic uptake, and is essential for maintaining the integrity of the micropore structure [68]. The Kelch13 complex is involved in the salvage of host cytosolic proteins together with other micropore proteins, and micropore maximal activity requires the ceramide de novo synthesis pathway. Unfortunately, the uptake of other nutrients by micropores was not observed, and the ability of micropores to uptake host nutrients by *T. gondii* needs to be further studied.

## Apicoplast transporters: bridging organellar metabolism

The apicoplast is a unique organelle thought to have a cyanobacterium origin [69, 70]. During evolution, the apicoplast lost its photosynthetic capacity and, consequently, the ability to fix carbon, generate ATP, and produce reducing power through photosynthesis. Nonetheless, it retains many essential metabolic activities. Metabolites synthesized within the apicoplast are partly utilized for its own maintenance, while others are exported to support other cellular compartments, including the mitochondria, cytoplasm, and endoplasmic reticulum (ER) [71–73]. Therefore, other metabolites from the metabolic chamber are also required to enter the apicoplast, including energy sources, carbon sources, reduction equivalents, cofactors, amino acids, and inorganic ions, in order to satisfy biosynthesis and maintain apicoplast activity [72, 74, 75]. The apicoplast is a four-membrane-enclosed organelle, which leads to great difficulties in material transport between the apicoplast and other metabolic compartments [76].

**Fig. 1:**
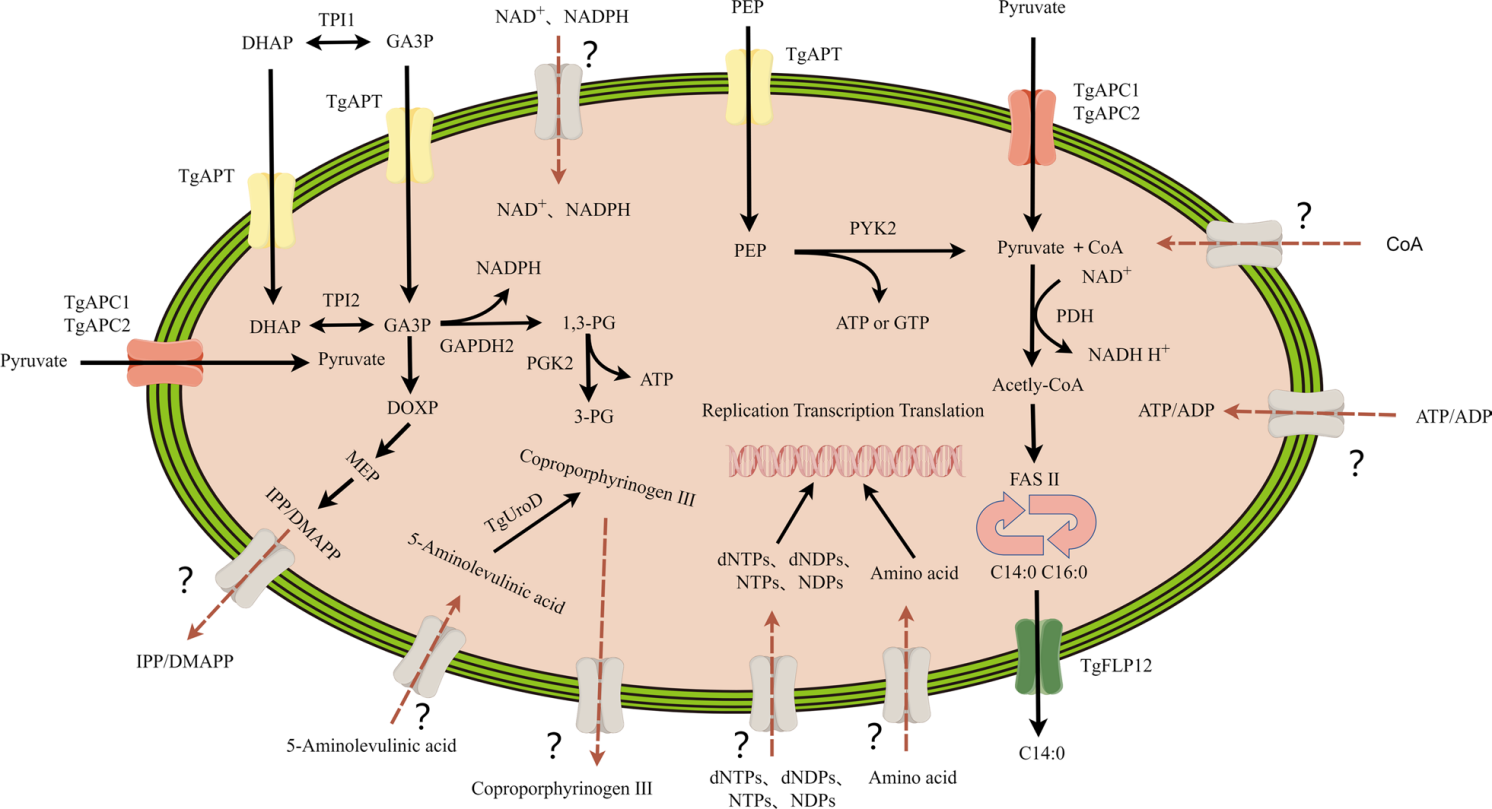
The transporter proteins of the apicoplast. The apicoplast of *T. gondii* requires a variety of transporters to maintain metabolic pathways and export metabolites from the apicoplast to the cytosol. *Tg*APT (yellow), *Tg*APC1/*Tg*APC2 (red), and *Tg*FLP12 (green) are the only three transporters that have been identified; they are phosphate and pyruvate transporters, respectively. In addition, it is speculated that there may unidentified transporters on the apicoplast that are responsible for transporting substrates into the apicoplast or delivery of the products from the apicoplast to the cytoplasm and other organelles. The gray modules in the figure represent potential transporters and their substrates

With continuous research, the metabolic pathways in apicoplasts have been basically determined. Well-studied pathways include the DOXP/MEP and FASII pathways. The DOXP/MEP pathway uses pyruvate and glyceraldehyde 3-phosphate as substrates to synthesize isopentenyl diphosphate (IPP) and its isomer dimethylallyl diphosphate (DMAPP), which are precursors for the synthesis of all isopentenoid substances [77, 78]. It is also a prosthetic group on many enzymes, a precursor to ubiquinone and polyphenols, and is involved in electron transport processes and glycoprotein formation [79]. Isoprene modifies proteins and maintains cell structure, and is necessary for almost all cells. Since the mevalonate pathway is used to synthesize IPP and DMAPP in the cytoplasm of host cells, the DOXP/MEP pathway can be used as an anti-parasitic drug target [80, 81]. In *T. gondii*, the FASII pathway is involved in the de novo synthesis of long-chain fatty acids such as myristate and palmitic acid, and fatty acids are the core components of cell membranes [72]. The majority (60–80%) of myristic acid and palmitic acid in *T. gondii* is derived from FASII, making the apicoplast FASII pathway an important source of cellular fatty acids [82]. Moreover, this pathway exists only in the apicoplast, and FASII has also long been considered a good drug target [77, 83]. So where do the substrates needed for the DOXP/MEP pathway and the FASII pathway (and the metabolites produced) come from and where do they go? The apicoplast is enclosed by four membranes, posing a substantial barrier to the constant flux of metabolites required for its maintenance and metabolic functions. Despite this demand, only a handful of apicoplast transporters have been identified to date. These include *Tg*APT (apicoplast phosphate translocator) and *Tg*APC/*Tg*AMT (apicoplast pyruvate carriers *Tg*APC1 and *Tg*APC2, also called apicoplast monocarboxylate transporters *Tg*AMT1 and *Tg*AMT2) (Fig. 1) [14, 74, 84, 85].

### Phosphate and pyruvate transport

At present, many studies have shown that the FASII pathway and DOXP/MEP pathway of *T. gondii* apicoplasts use pyruvate and glyceraldehyde 3-phosphate (GA3P, also called triose phosphate) as substrates [14, 73, 77, 78]. *Tg*APT was the first identified transport protein (phosphate translocator) in *T. gondii* apicoplasts that linked cytosolic and apicoplast metabolism [84]. In *T. gondii* apicoplasts, *Tg*APT imports glycolytic intermediates from the cytosol, including GA3P, phosphoenolpyruvic acid (PEP) and dihydroxyacetone phosphate (DHAP); the genetic ablation of *Tg*APT results in the rapid death of parasites [84, 86]. Besides, four glycolytic enzymes, triosephosphate isomerase 2 (TPI2), glyceraldehyde phosphate dehydrogenase (GAPDH2), pyruvate kinase 2 (PYK2), and phosphoglycerate kinase 2 (PGK2), were utilized to synthesize key substrates of the DOXP/MEP and FASII pathways in *T. gondii* apicoplasts. TPI2 in apicoplasts converts DHAP to GA3P, providing a substrate for the DOXP/MEP pathway.

Given that TPI2 is essential for in vitro tachyzoite proliferation, genetic disruption of this apicoplast-localized enzyme impairs isoprenoid precursor biosynthesis through the methylerythritol phosphate (MEP) pathway, ultimately resulting in lethal growth defects [77]. This suggests that GA3P transported by *Tg*APT into the apicoplast does not fully meet the needs of the DOXP/MEP pathway in *T. gondii*, and that conversion of DHAP to GA3P by TPI2 is a more important source in the apicoplast.

PYK2 is the only known enzyme that catalyzes PEP to product pyruvate in apicoplasts, and PEP in apicoplasts is transported by *Tg*APT. However, the absence of PYK2 has no obvious effect on parasite growth. It is speculated that there may be transporters that transport pyruvate in apicoplasts [77]. Recent investigations have identified two apicoplast membrane-localized proteins, *Tg*APC1 and *Tg*APC2, which form a functional apicoplast pyruvate carrier (APC), which is essential for cytosolic pyruvate translocation. Genetic ablation of this heterodimeric transporter induces marked attenuation of apicoplast metabolic pathway activities and organellar structural compromise, consequently triggering complete proliferative arrest in tachyzoites [74]. However, there are some confusing problems in the existing findings. For example, quantitative profiling of apicoplast metabolism revealed that genetic disruption of either APC1 or APC2 suppresses FASII biosynthetic flux by 50%, with concomitant reductions in IPP/DMAPP and MEP pathway intermediates to residual levels of 5–17% [74]. These findings collectively demonstrate that APC1/2 ablation predominantly compromises the FASII pathway while more modestly impacting isoprenoid precursor production via the MEP route. However, the latest research results show that the FASII pathway is dispensable during the lytic cycle of *T. gondii*, because the pyruvate dehydrogenase complex (PDC) and FabD (malonyl-CoA- [acyl carrier protein] transacylase), which are located in the apicoplast and drive de novo fatty acid biosynthesis, can tolerate the genetic deletions [72]. Therefore, the mechanism of pyruvate transporter deficiency causing parasite death still needs to be further explored. Interestingly, APC1 and APC2 were also examined in depth in a study published almost simultaneously, in which they were named AMT1 and AMT2, respectively. The results of the two studies were almost consistent, protein depletion of AMT1 and AMT2 resulted in defects in plaque formation in vitro, ACP diffusion/apicoplast loss, and MEP and FASII biosynthetic pathways [85]. Unfortunately, the attempt to identify the transported substrates of *Tg*AMT1 and *Tg*AMT2 was not successful. However, systematic integration of proximity-dependent biotinylation and CRISPR-Cas9-mediated endogenous tagging has delineated a cohort of previously uncharacterized transporters within the apicoplast membrane system [85].

Fatty acid synthesis in apicoplasts provides substrate for a large amount of lipid synthesis in the ER [87–89]. Therefore, it is speculated that there may be transporters transporting fatty acids on the apicoplast. In the latest research, researchers identified a completely new lipid transporter protein *Tg*FFLP12, which belongs to the p5-ATP enzyme transporter, which is located in the apicoplast of *T. gondii* [90]. The researchers conducted a comprehensive study on the lipid profile of parasites lacking *Tg*FLP12, and found that it was of great significance for the export of the short fatty acid chain myristic acid (C14:0) from the apicoplast. *Tg*FLP12 depletion reduces *Toxoplasma* division rate after complete loss of the protein and reduces parasite fitness in the long term. Disruption of *Tg*FLP12 causes major defects in apicoplast morphology. Lipidomic analyses and stable isotope labeling reveal a unique accumulation of C14:0 in the apicoplast, which is then lacking in most major lipid classes subsequently synthesized in the ER [90].

### Unresolved questions

In addition to the apicoplast transporters that have been identified so far, the substrate requirements and energy requirements of the apicoplast suggest that other transporters may be present on the apicoplast. PYK2 and PGK2 are the only enzymes known to produce energy in apicoplasts; however, loss of both PYK2 and PGK2 has no significant effect on parasite growth. Therefore, there may be other sources of energy required for metabolic pathways in the apicoplast, and it is speculated that there may be ATP transporters on the apicoplast [14, 77]. PYK2 in *T. gongii* apicoplasts tend to use GDP as substrate rather than ADP as substrate, as dNTP and NTP cannot be synthesized in apicoplasts, thus they lack the raw materials for genome replication and transcription; the source of proteins in the apical plastid is unclear. Besides, in plant plastids, nucleotide transporters are known to exist in chloroplasts, so it is hypothesized that nucleotide transporters also exist in *T. gondii* apicoplasts to transport nucleotide raw materials into the apicoplast to synthesize these proteins [91]. Apicoplasts require both NADH and NADPH forms of reducing power; however, the origin of reductive NAD^+^ and NADP^+^ in apicoplasts remains unclear [92–95]. In apicomplexan parasites, NADH is produced by the pyruvate dehydrogenase complex (PDH) located in the apicoplast; however, PDH is not required in tachyzoites and hematogenous *Plasmodium* [96]. Therefore, this may not be the only source of NADH, and it is speculated that there may be transporters on apicoplasts that transport cytoplasmic NADH into the apicoplast. GA3P can be used as a substrate of GAPDH2 to produce 1,3-PG (1,3-diphosphoglyceric acid) and NADPH, from which the reducing power required for many metabolic pathways in the apicoplast is derived; however, GAPDH2 is dispensible in *T. gondii* [77]. Besides, in the apicoplast of *T. gondii*, there is isocitrate dehydrogenase 1 (ICDH1), which can catalyze the production of NADPH. However, the absence of ICDH1 only causes moderate growth defects in *T. gondii* rather than death [97]. Therefore, it is speculated that there is a transporter that transports NADPH from the cytoplasm to the apicoplast. Besides, the lack of the PDH-E1α subunit leading to growth defects was improved by supply of exogenous fatty acids, suggesting that *T. gondii* can salvage fatty acids from the environment, although the detailed mechanisms warrant further investigation [72]. Proteins are made of amino acids, but the origin of the proteins in apicoplasts remains unclear. A few amino acids can be synthesized in the cytoplasm and mitochondria of *T. gondii*, and there are amino acid transporters in the plasma membrane, which can absorb amino acids from the host into the cytoplasm of *T. gondii*. These amino acids may enter the apicoplast through the transporters on the apicoplast, thus forming the apicoplast proteins. The FASII pathway also requires many other metabolic substances, such as ADP, ATP, GDP, NAD^+^/NADPH, coenzyme A (CoA)/dephospho-coenzyme A, *S*-adenosine methionine (SAM), thiamine, and biotin [98]; however, their sources are unknown. Dephospho-CoA kinase (DPCK) is the last enzyme in the coenzyme A synthesis pathway [99]. CoA is essential for fatty acid synthesis in apicoplasts [98, 100], whereas *T. gondii* DPCK is located in the cytoplasm, it is therefore speculated that CoA in apicoplasts comes from the cytoplasm, and there may be CoA-related transporters on apicoplasts [101]. IPP and DMAPP are isoprene precursors produced in apicoplasts, which produce geranylpyrophosphate (GPP), farnesyl diphosphate synthase (FPP), and geranylgeranyl diphosphate (GGPP), as well as long chain isoprene in the cytoplasm, so it is speculated that there are transporters to transport them to the cytoplasm, and thus may be ideal drug targets [77]. Heme is a ubiquitous molecule that functions in a variety of essential life processes [102, 103]. In *T. gondii*, heme biosynthesis initiates in the mitochondrion with the production of 5-aminolevulinic acid [103]. This precursor is then imported into the apicoplast, where it is converted to coproporphyrinogen III through the catalytic actions of four enzymes: 5-aminolevulinate dehydratase (ALAD), porphobilinogen deaminase (PBGD), uroporphyrinogen III synthase (UROS), and uroporphyrinogen decarboxylase (UROD) [56, 104]. Coproporphyrinogen III is subsequently exported to the cytosol for further modification before re-entering the mitochondrion to complete the final steps of heme synthesis. This process in apicoplasts is currently proposed to require two transporters: one to import the substrate, 5-aminolevulinic acid, from the cytosol into the apicoplast, and another to export the resulting intermediate, coproporphyrinogen III, from the apicoplast back to the cytosol. However, the identities of these two transporters have not yet been identified [105, 106].

## Mitochondrial transporters: powerhouses and metabolic hubs

Apicomplexa phylum parasites have two organelles of endosymbiotic origin, apicoplasts and mitochondria, which are important metabolic chambers for many metabolic processes in apical complex parasites. Apicoplasts and mitochondria are closely related physically, and some important metabolic pathways require the collaboration of the two organelles, such as the heme metabolism pathway, the FASII pathway and the tricarboxylic acid (TCA) cycle [105, 107, 108].

### Mitochondrial pyruvate carriers are dispensable for *Toxoplasma gondii*

*Toxoplasma gondii* displays metabolic plasticity during proliferation, with demonstrated competence in utilizing diverse carbon substrates. The maintenance of pyruvate homeostasis emerges as an essential determinant for the survival of *T. gondii*. This pivotal metabolite is synthesized predominantly in the cytosol yet catabolized through specialized pathways within discrete subcellular compartments, including mitochondria and the apicoplast [82]. Acetyl-CoA is necessary for the acetylation of proteins, DNA, and RNA, and more importantly, mitochondrial acetyl-CoA can drive energy production. Some studies have found that mitochondrial acetyl-CoA mainly comes from pyruvate in the cytoplasm, which can be converted to lactic acid and amino acids in the cytoplasm. Mitochondria can catalyze branched-chain ketoate dehydrogenase (BCKDH) to produce acetyl-CoA (CoA) for the TCA cycle [109]. Studies have shown that two mitochondrial pyruvate carriers (MPC1 and MPC2), were identified to transport pyruvate from the cytoplasm to the mitochondria [75]. However, MPC1 and MPC2 have been shown to be expendable for *T. gondii* growth through gene knockout studies, suggesting that there may be other sources of pyruvate or acetyl-CoA in the mitochondria.

In mammalian cells, alanine can enter the mitochondria, where it is converted to pyruvate by glutamic-pyruvic transaminase 2 (GPT2) [110–112]. GPT2 homologs have been found in the *T. gondii* genome, suggesting the presence of alanine transporters on the mitochondria of *T. gondii*, but further studies are needed. In other organisms, β-oxidative metabolism is an important source of acetyl-CoA in mitochondria, but the potential contribution of mitochondrial β-oxidative metabolism to acetyl-CoA pools remains uncharacterized in *T. gondii*. However, mitochondrial acyl-carnitine/carnitine carriers are absent in *T. gondii* and the expression of β-oxidizing proteins in tachyzoites was low, suggest that fatty acid β-oxidative metabolism may not be a source of acetyl-CoA in *T. gondii* mitochondria [75, 113].

### Fe–S cluster intermediate transporters are essential for electron transport in* T. gondii* mitochondria

Iron–sulfur (Fe–S) clusters, serving as widespread inorganic cofactors, constitute essential structural motifs in enzymatic complexes that orchestrate indispensable cellular processes, and they also mediate electron transport reactions and catalysis in many critical processes [114, 115]. Fe–S clusters in cells cannot be obtained from the environment and can only be synthesized by the cells themselves using iron ions (Fe^2+^ or Fe^3+^) and sulfur ions (S^2−^). Fe–S clusters have a wide range of functions in cells, and mitochondria are one of the most important organelles for the synthesis of Fe–S clusters. The mitochondrial iron–sulfur cluster assembly pathway (the ISC pathway), the plastid sulfur mobilization pathway (the SUF pathway), and the cytosolic iron–sulfur assembly pathway (the CIA pathway) together constitute the synthesis pathway of Fe–S clusters in cells [116]. In mitochondria, mitochondrial cysteine desulfurase (NFS1) releases sulfur from cysteine formed from Fe–S cluster intermediates, which then binds to iron ions and assemble into Fe–S clusters on the mitochondrial scaffold protein ISU1 [117, 118]. In addition to the ISC pathway, the CIA pathway also depends on iron–sulfur cluster intermediates produced in the mitochondria by NFS1. Thus, the cytosolic CIA pathway requires mitochondrial export of sulfur-containing intermediates to function. The earliest Fe–S cluster intermediate transporter was discovered in yeast, named ATP-binding cassette (ABC) transporter ABCB7 (ATM1), which functions in a glutathione-dependent manner. In plants and mammals, the Fe–S cluster intermediate transporters have also been identified, namely, ATM3 and ABCB7, respectively [119, 120]. In the Apicomplexa phylum, Fe–S cluster intermediate transporters have only recently been intensively studied. In two recently published contemporaneous studies, *TG*GT1_269000 was identified as the putative *T. gondii* ATP-binding cassette (ABC) transporter ABCB7 (*Tg*ATM1 or *Tg*ABCB7L) [121, 122]. Both studies suggest that *Tg*ATM1 localizes in mitochondria and is essential for *T. gondii* growth, but depletion of *Tg*ATM1 does not specifically impair mitochondrial metabolism. Proteomics and metabolomics analysis showed that the depletion of *Tg*ATM1 leading to Fe–S protein defects, and endogenous expression of *Sc*ATM1 was able to completely rescue the phenotypic defects caused by *Tg*ATM1 depletion [122].

### Energy produced by mitochondria requires specific transporters to transport it to the cytoplasm

Energy production is one of the important functions of mitochondria and mainly exists in the form of ATP. Previous studies have shown that the mitochondrial membrane, including other cytoplasmic membranes, does not allow ATP and ADP to shuttle freely, and requires specific ATP/ADP transporters. Mitochondrial ADP/ATP vectors *Tg*AAC1 and *Tg*AAC2 (ADP/ATP carriers) have been found in *T. gondii*, and the expression of *Tg*AACs in *Escherichia coli* cells shows that only *Tg*AAC1 can transport ATP. In addition, *Tg*AAC1 conditional knockout experiments showed severe growth defects in *T. gondii*, and *Tg*AAC1 growth defects were recovered in allogeneic supplementation mice with *Tg*AAC1 deletion mutants, indicating that *Tg*AAC1 is essential for *T. gondii* [123, 124].

### Unresolved problems

In addition to the above studied transporters, current studies have shown that there are many mitochondrial transporters, ion channels, or shuttle channels in yeast and mammals, these transporters have not been studied in *T. gondii* or Apicomplexa phylum parasites. The TCA cycle is an important metabolic pathway in mitochondria, in addition to the known pyruvate transporter, succinate/fumarate carriers also exist in yeast, which transport fumarate from mitochondria to the cytoplasm and an equivalent amount of cytosolic succinate to mitochondria [125, 126]. In *S. cerevisiae*, the citrate/α-ketoglutarate carrier plays a central role in exporting acetyl-CoA as citrate from the mitochondria to the cytoplasm, which in turn links carbohydrate catabolism and lipogenesis [127]. Other more important transporters such as amino acid mitochondrial carriers [128] and mitochondrial cofactor carriers [129–131] have not been studied in *T. gondii*.

## ER and Golgi transporters: secretory pathway gateways

In eukaryotic cells, proteins, particularly secretory and membrane-bound proteins, must be processed and folded within the ER, where they undergo post-translational modifications such as glycosylation and acetylation. Concurrently, the ER serves as a key site for fatty acid elongation [49, 132, 133].

Acetyl-CoA is a key substance in a variety of metabolic pathways in *T. gondii*. The predominant acetyl-CoA in *T. gondii* is produced from glycolysis-derived pyruvate, which is catalyzed by BCKDH and recycled by the TCA cycle in mitochondria [109]. In *T. gondii*, there is an apicoplast-derived pyruvate dehydrogenase (PDH) that catalyzes pyruvate to acetyl-CoA, the major precursor of the FASII pathway [14, 72, 134]. The synthesis of FAS is carried out by the sequential addition of acetyl-CoA-derived two-carbon units, and the FASII pathway in *T. gondii* takes place in the apicoplast. In addition, *T. gondii* can also use exogenous acetate to synthesize acetyl-CoA under the acetate-CoA synthetase in the cytoplasm, which is the main source of acetyl-CoA in the ER of *T. gondii* [135]. Apicoplast-generated FAs are then exported toward the ER and functions with acetyl-CoA as a common substrate for the production of complex fatty acids by hydroxyacyl-CoA dehydratase and enoyl-CoA reductase enzymes. It is used to maintain lipid homeostasis in each organelle of the parasite [136]. In addition, acetyl-CoA is the only acetyl donor of secreted protein acetylation modification in the ER. Although acetyl-CoA plays an important role in the ER, how it enters the ER is not well understood.

### The acetyl-CoA transporter in the ER has not been characterized

Acetyl-CoA, a membrane-impermeant metabolite formed by the conjugation of an acetyl group with coenzyme A, is actively transported into the ER lumen in mammalian cells via the acetyl-CoA transporter (AT1, also known as SLC33A1) [137, 138]. In the Apicomplexa phylum, the ER acetyl-CoA transporter has not been identified. During drug-resistant genetic screening process, two novel *Plasmodium* drug resistance genes located in the ER were identified, including a putative acetyl-CoA transporter (*Pf*ACT, PF3D7_1036800), as well as a putative UDP-galactose transporter (*Pf*UGT, PF3D7_1036800) [139]. Subsequently, the acetyl-CoA transporters in *P. berghei* and *T. gondii* were identified by homology analysis with mammalian AT1, and named as *Pb*AT1 (PBANKA_0519800) and *Tg*AT1 (*TG*ME49_215940), respectively [140]. By homology analysis, we found that the amino acid similarity between *Pf*ACT and *Pb*AT1 was about 80%, indicating that they are homologous proteins. Unfortunately, neither *Pf*ACT, *Pb*AT1, nor *Tg*AT1 have been validated for their acetyl-CoA transport capacity, and they are still putative acetyl-CoA transporters. In addition, acetyl-CoA as a substrate of secreted protein acetylation, global acetylome profiling of both in both *T. gondii* and *P. berghei* AT1 knockout parasites only revealed modest or even no alteration of the acetylomes of secretory proteins [140]. This suggests that *Tg*AT1 and *Pb*AT1 may not be the ER acetyl-CoA transporters of *T. gondii* and *P. berghei*, or there are multiple acetyl-CoA transporters in *T. gondii* and *P. berghei* that work together to maintain a steady-state of acetyl-CoA content in the ER. Recent studies show that *Tg*AT1 contributes to fatty acids synthesis in the parasite. Although *Tg*AT1 deletion reduced ^13^C incorporation into fatty acids, it did not abolish it completely. This partial effect aligns with fatty acids in *T. gondii* being initiated by the FASII de novo synthesis pathway within the apicoplast, a process not reliant on *Tg*AT1 function [141]. In summary, there is no direct evidence to demonstrate that *Tg*AT1 is an acetyl-CoA transporter in the ER of *T. gondii*.

### The ER of *T. gondii* also requires an adequate energy supply

The secretory proteome of *T. gondii* is required for host-cell invasion and intracellular proliferation and undergoes ER dependent biosynthesis before targeted transport to specific organelles, including apicoplasts, micronemes, dense granules, and rhoptries. Secretory proteins are energy dependent and require chaperone proteins for their synthesis in the ER [142–144]. Numerous studies have shown that ATP cannot directly cross phospholipid bilayers such as cell membranes, mitochondrial membranes, and ER membranes. Therefore, specific transporters are required to transport ATP from the cytosol to the ER, and the reaction product, ADP, out of the ER [145–147]. In our previous study, we successfully identified the first ATP/ADP transporters *Tg*ANT (ATP/ADP transporter) and *Pf*ANT in *T. gondii* and *P. falciparum*, respectively [148]. Deletion of *Tg*ANT causes death of *T. gondii*, and mutations in its two key domains also cause fatal phenotypic defects. Previous studies showed that a single amino acid mutation (F37V) in *Pf*ANT resulted in marked resistance to imidazolopiperazines [139]. Competition experiments showed that *Pf*ANT also has a certain transport capacity for imidazolopiperazines, and its presence can inhibit the uptake of α-^32^P–ATP [148]. Combining previous research results, we speculated that imidazolopiperazine drugs may transport into the ER by *Pf*ANT, and its amino acid mutation results in the decrease of drug transport capacity, which then leads to drug resistance, but further verification is needed. In addition, because the domain of the amino acid mutation that causes resistance in *Pf*ANT is fully conserved in *Tg*ANT, it is necessary to investigate whether the same resistance mechanism exists in *T. gondii*.

### Nucleotide sugar transporters: key players in* T. gondii* glycoconjugate biosynthesis

Another important function of the ER is protein glycosylation, including *N*-glycosylation, *O*-glycosylation, and *O*-fucosylation, etc. The glycosylation of these proteins occurs in the ER or Golgi apparatus, or both for protein glycosylation modification [149–151]. Glycoconjugates on the cell surface of apicomplexan parasites play key roles in determining parasite–host interactions and survival [151–153].

In eukaryotes, *N*-linked glycosylation of proteins initiates in the ER and follows a conserved biosynthetic pathway. This process requires the assembly of an asparagine-linked oligosaccharide precursor (lipid-linked oligosaccharide, LLO), which is subsequently modified by a series of glycosyltransferases to generate complex glycan structures [154]. The oligosaccharide is then transferred “en masse” to the polypeptide backbone by oligosaccharide transferase (OST), where the modification site is the Asn residue containing the N–X–S/T sequence on the protein. The transfer of *N*-acetylgalactosamine (GalNAc) to the hydroxyl group of a specific serine (Ser) or threonine (Thr) residue initiates *O*-GalNAc glycosylation modification, which is a common post-translational modification of secreted or membrane-associated proteins in eukaryotes. Unlike *N*-glycosylation, which begins in the ER and ends in the Golgi apparatus, *O*-glycosylation occurs only in the Golgi apparatus [155, 156]. This type of glycosylation is also known as mucin-type *O*-glycosylation and controls the chemical, physical, and biological properties of mucin [150]. Although both *T. gondii* and *Plasmodium* belong to the phylum Apicomplexa, there are huge differences in *N*-glycosylation between them. For example, it is generally believed that the *N*-glycosylation modification of *Plasmodium* is very simple, and its genome lacks a series of glycosyltransferase genes, and can only be modified by 1,2–*N*-acetylglucosamine (GlcNAc) [157, 158]. *T. gondii*, however, is capable of the same complex *N*-glycosylation of proteins as mammalian cells, but the glycan structure formed in the ER is still controversial. It has even been shown that *T. gondii* can ingests glycan structures from the host [157, 159, 160]. Both *N*-linked and *O*-linked glycosylation require nucleotide sugar-activated monosaccharides as glycosyl donor substrates for glycan biosynthesis. As nucleotide sugars are primarily utilized by glycosyltransferases in the ER and/or Golgi apparatus, they must be translocated into these organelles by specific transporters. However, there are few studies on nucleotide sugar transporters in *T. gondii*, and only some putative nucleotide sugar transporters exist; only two monosaccharide nucleotide transporters have been identified so far.

The *Toxoplasma gondii* nucleotide-sugar transporter (*Tg*NST1) was the first nucleotide-sugar transporter to be reported that can transport UDP-GlcNAc and UDP-GalNAc across membranes, and this transporter aids in the persistence of *T. gondii* tissue cysts in animals [153]. In vitro, deletion of *Tg*NST1 specifically impairs glycosylation of cyst wall components, leading to structural disruption of the cyst wall, and in vivo, this deletion severely compromises the parasite’s ability to establish chronic infection. These results demonstrate, for the first time, the critical role of the glycosylation substrate transporter in *T. gondii*. Unfortunately, the transport activity of *Tg*NST1 was verified through heterologous expression, and there is no direct evidence to prove that they are UDP-GlcNAc and UDP-GalNAc transproteases. Moreover, there is currently controversy regarding its subcellular localization [153]. Using the human Golgi GDP–Fuc transporter as a template, a BLASTP search identified a single putative GDP-sugar transporter, *Tg*GT1_267730 (designated NST2). MIC2, a highly glycosylated microneme protein in *T. gondii*, undergoes *O*-fucosylation, and the disruption of NST2 results in a loss of MIC2 *O*-fucosylation and a marked reduction in MIC2 cellular levels. This phenotypic defect of MIC2 glycosylation deficiency is consistent with the MIC2 glycosylation defect caused by deletion of POFUT2 (*O*-fucosyltransferase 2). Consequently, these findings support the identification of NST2 as a GDP–Fuc transporter involved in *O*-fucosylation within the parasite [151]. But, as with NST1, there is no direct evidence that NST2 is a GDP–Fuc transporter.

## A systematic approach to transporter discovery

Although some transporters have been identified in *T. gondii*, there are still many transporters that have not been found and identified. In particular, some transporters with important functions may be promising drug targets, so it is essential to screen, find, and research these potential transporters. In general, *T. gondii* transport proteins have multiple transmembrane domains (TMD), of course, some membrane-bound proteins such as transferases may also have multiple TMD. In addition, localizations of thousands of proteins in *T. gondii* have been predicted by applying the hyperplexed localization of organelle proteins by isotope tagging (hyperLOPIT) [161]. Therefore, transporters in *T. gondii* were screened by setting the TMD value (≥ 6) and CRISPR phenotype value (≤ −3) [50] in *ToxoDB*[34] (https://toxodb.org/toxo/app/), and a total of 69 potential *T. gondii* transporters were identified. Then, the screened potential transporters were matched in the hyperLOPIT database and the localization of these proteins was determined. Subsequently, we searched the potential transporters in the National Center for Biotechnology Information (NCBI), and summarized the locations and functions of the transporters that had been studied (Additional file 1). Among the screened proteins, there were several transporters that were identified (gray background) and known to be important for *T. gondii*, such as an amino acid transporter (*Tg*ApiAT2) [7], an ER ATP/ADP transporter (*Tg*ANT) [148], and an apicoplast phosphate transporter (*Tg*APT1) [86]. It also contains some membrane-bound proteins and enzymes (green background) such as phosphatidate cytidylyltransferase (*Tg*CDS1) [162]. Therefore, we believe that screening using TMD and CRISPR phenotypic values, combined with protein localization data from hyperLOPIT, can effectively search for functionally important new transporters in *T. gondii*. In addition, this method has been used to screen transporters on organelles, which is able to effectively narrow the range of target transporters [141, 148]. While the individual components of this strategy are well-established, their integration provides a systematic framework to effectively narrow the candidate list and prioritize transporters for functional validation, it is important to note that while the hyperLOPIT dataset is an invaluable resource for predicting protein localization, it may contain inaccuracies, as evidenced by the initial mislocalization of the apicoplast transporters AMT1 and AMT2 [85]. Therefore, the subcellular localization of the transport proteins obtained through this screening approach still requires further confirmation through methods such as endogenous tagging.

## Concluding remarks and future directions

Much work remains to be done on the transporters of *T. gondii* (Additional file 2). In *Plasmodium*, 26 transporters were found to be implicated in the drug resistance to existing antimalarials and/or drugs within the development pipeline, indicating that transporters are likely to serve as drug targets not only in in *Plasmodium* but also in *T. gondii*. Although transporters are promising drug targets, there are some urgent problems to be solved in the process of transporter research, which hinders further research on transporters. The main bottleneck in transporters research is how to determine transport activity, and key to this work is the establishment of heterologous expression systems, thereby elucidating the activity of transporters in vitro and differentiating them from other membrane-bound proteins and enzymes. Commonly used heterologous expression systems include the *E. coli* expression system, the yeast expression system, and the *Xenopus* oocyte system. Several recent studies on the substrate specificity of *T. gondii* transporters were completed in *E. coli*. However, there are some problems in the *E. coli* expression system, such as the lack of efficient membrane protein expression function assays and insufficient post-translational modifications such as glycosylation or phosphorylation, etc., which make it difficult to express exogenous membrane proteins in *E. coli* membranes or results in incorrect simulation of the conformation of exogenous membrane proteins. The *Xenopus* oocyte expression system and the yeast expression system are the most suitable for expressing *T. gondii* transporters, as both expression systems are also amenable to the production and purification of transporters for structural determinations, following the use of cryo-electron microscopy, allowing for high-resolution protein conformations of the transporters. Another barrier is how to ascertain the transport substrates of transporters, and then select the appropriate substrates among many metabolites to verify the transport activity. At present, most of the annotated transporters in the *T. gondii* database are putative proteins; the potential transport substrates cannot be determined. Combining the subcellular localization assays of transporters with targeted or nontargeted metabolomics analysis of gene deletion strains may allow new breakthroughs in studies of transport properties.

In summary, although many membrane transporters of *T. gondii* have been identified, studies have shown that *T. gondii* can scavenge more nutrients from the host cell, including but not limited to host proteins, fatty acids, myo-inositol, serine, ethanolamine, choline, ceramide, sphingolipids, cholesterol, vitamins, and cofactors [6, 104, 163–169], but the transport mechanisms for these substances remains unclear. These substances will be a new direction for future research on *T. gondii* transporters.

## Supplementary Information

Supplementary material 1. Additional file 1. Potential transporters screened out from ToxoDB

Supplementary material 2. Important transporters and their functions of *Toxoplasma gondii*

## Data Availability

All data generated or analyzed during this study are included in this published article and its supplementary information files.
