# Supplementary material for: Transporters, an important but poorly studied area of Toxoplasma gondii
Source: Parasit Vectors. 2026 Jan 25;19:51. doi: 10.1186/s13071-025-07216-w (PMC12849547; doi:10.1186/s13071-025-07216-w)
Supplement: Supplementary file 2 — Supplementary material 2. Important transporters and their functions of Toxoplasma gondii [file 13071_2025_7216_MOESM2_ESM.docx]

Additional file 2. Important transporters and their functions of *Toxoplasma gondii*

| Name | Gene  number | Location | Substrate | Function | References |
| --- | --- | --- | --- | --- | --- |
| TgGT1 | TGME49_214320 | Plasma membrane | Glucose、Mannose、 Galactosefructose | Transport glucose、 mannose、galactose and fructoset， provide carbon source for energy metabolism in *T. gondii* | [1, 2] |
| TgST1, TgST3 | TGGT1_257120  TGGT1_201260 | Intracellular vesicles | Unannotated | Unannotated | [1] |
| TgST2 | TGME49_272500 | Plasma membrane | Unannotated | Unannotated | [1] |
| TgPiT | TGGT1_240210 | Mainly in the plasma membrane (also in inner buds of endosomal VAC organelles and some cytoplasmic vesicles) | Pi、ATP、AMP | Absorb inorganic phosphate with Na^+^ gradient | [3, 4, 5] |
| TgPT2 | TGGT1_235150 | Plasma membrane | Pi、ATP、AMP | Absorb inorganic phosphate and maintain the growth, movement and invasion ability of insect body | [5] |
| TgmPT | TGGT1_278990 | Mitochondria | Unannotated | Unannotated | [5] |
| TgApiAT5-3 | TGME49_257530 | Plasma membrane | L-tyrosine、Aromatic amino acid、Neutral amino acid | Promote the transport of L-Tyr into Toxoplasma, and maintain the concentration of aromatic amino acids and large amounts of neutral amino acids in the cell through exchange, so as to maintain the growth and virulence of *T. gondii* | [6, 7] |
| TgApiAT1/TgNPT1 | TGME49_215490 | Plasma membrane | Arginine | Transport arginine, mediate amino acid exchange, maintain the growth and virulence of *T. gondii.* | [8] |
| ApiAT6-1 | TGME49_240810 | Plasma membrane | Lysine、Arginine、many Cationic amino acids、 large neutral amino acids and Arginine | Mediates high affinity uptake of lysine and low affinity uptake of arginine, mediates amino acid exchange, and maintains the growth and virulence of *T. gondii* | [8, 9] |
| TgAT1 | TgGT1_244440 | Plasma membrane | Tritiated adenosine、 Inosine、Hypoxanthine and Adenine | Transport adenosine and inosine | [10, 11] |
| TgAT2 | Uncommented | Plasma membrane | Adenosine、Inosine、Guanosine、 Uridine、Thymine and Cytidine | Transport all natural purines and pyrimidines | [10] |
| TgNBT1 | Uncommented | Plasma membrane | Hypoxanthine、Xanthine and Guanine | Transport hypoxanthine,、xanthine and guanine | [10] |
| TgFNT1、TgFNT2 TgFNT3 | TGGT1_209800  TGGT1_292110  TGGT1_229170 | Plasma membrane | L-lactic acid、formic acid | Transport L-lactic acid and formic acid in a ph-dependent manner | [12] |
| TgATP4 | TGME49_278660 | Plasma membrane | H^+^、Na^+^ | At the same time, H^+^ was transformed inward and Na^+^ outward to maintain the homeostasis of Na^+^, maintain the growth of the extracellular worm and the virulence of the worm in vivo | [13] |
| TgTRPPL-2 | TGGT1_310560 | Plasma membrane and ER | Ca^2+^ | Ca^2+^ uptake into tachyzoite cytoplasm and Ca^2+^ expulsion from the endoplasmic reticulum of tachyzoites maintain the invasion and overflow of insect bodies and maintain the growth of insect bodies | [14] |
| TgAPT | TGGT1_261070 | Apicoplast | GA3P、PEP、DHAP | Glycolytic intermediates, including GA3P, PEP and DHAP, are imported from the cytoplasm to maintain the survival of the *T. gondii* | [15, 16] |
| TgAPC1/  AMT1, TgAPC2/  AMT2 | TGGT1_233540  TGGT1_297245 | Apicoplast | Pyruvate | Mediates the input of cytoplasmic pyruvate and maintains the activity of apicoplast metabolic pathway and the integrity of organelles | [17, 18] |
| MPC1, MPC2 | TGGT1_235880  TGGT1_204370 | Mitochondria | Pyruvate | Transport pyruvate from the cytoplasm to the mitochondria | [19] |
| TgFLP12 | TGGT1_289070 | apicoplast | apicoplast fatty | mediates apicoplast fatty export in Toxoplasma | [20] |
| TgAAC1 | TGGT1_249900 | Mitochondria | ATP | Transport ATP and maintain the growth of insect body | [21] |
| TgATM1/TgABCB7L | TGGT1_269000 | Mitochondria | Fe-S cluster intermediates | Fe-S cluster intermediates are exported from mitochondria to maintain DNA and RNA replication, protein translation and other metabolic pathways in the nucleus and cytoplasm, and maintain the growth of insect body | [22, 23] |
| TgAT1 | TGME49_215940 | ER | Unannotated | Putative acetyl-CoA transporter | [24] |
| TgANT | TGGT1_254580 | ER | ATP、ADP | Reverse transport of ATP/ADP can maintain the growth, replication and invasion of insect body | [25] |
| TgNST1 | TgME49_067380 | ER or Golgi apparatus | UDP-GlcNAc and UDP-GalNAc | Reverse transport of ATP/ADP can maintain the growth, replication and invasion of insect body | [26] |
| TgNST2 | TGGT1_267730 | Unknown | GDP-Fuc | Transport GDP-Fuc to maintain O-focusing of MIC2 | [27] |

1. Blume M, Rodriguez-Contreras D, Landfear S, Fleige T, Soldati-Favre D, Lucius R, et al. Host-derived glucose and its transporter in the obligate intracellular pathogen Toxoplasma gondii are dispensable by glutaminolysis. Proc Natl Acad Sci U S A. 2009;106 31:12998-3003; doi: 10.1073/pnas.0903831106.

2. Joët T, Holterman L, Stedman TT, Kocken CH, Van Der Wel A, Thomas AW, et al. Comparative characterization of hexose transporters of Plasmodium knowlesi, Plasmodium yoelii and Toxoplasma gondii highlights functional differences within the apicomplexan family. Biochem J. 2002;368 Pt 3:923-9; doi: 10.1042/bj20021189.

3. Saliba KJ, Martin RE, Bröer A, Henry RI, McCarthy CS, Downie MJ, et al. Sodium-dependent uptake of inorganic phosphate by the intracellular malaria parasite. Nature. 2006;443 7111:582-5; doi: 10.1038/nature05149.

4. Asady B, Dick CF, Ehrenman K, Sahu T, Romano JD, Coppens I. A single Na+-Pi cotransporter in Toxoplasma plays key roles in phosphate import and control of parasite osmoregulation. PLoS Pathog. 2020;16 12:e1009067; doi: 10.1371/journal.ppat.1009067.

5. Cui J, Yang X, Yang J, Jia R, Feng Y, Shen B. A Coccidia-Specific Phosphate Transporter Is Essential for the Growth of Toxoplasma gondii Parasites. Microbiol Spectr. 2022;10 5:e0218622; doi: 10.1128/spectrum.02186-22.

6. Parker KER, Fairweather SJ, Rajendran E, Blume M, McConville MJ, Bröer S, et al. The tyrosine transporter of Toxoplasma gondii is a member of the newly defined apicomplexan amino acid transporter (ApiAT) family. PLoS Pathog. 2019;15 2:e1007577; doi: 10.1371/journal.ppat.1007577.

7. Rajendran E, Hapuarachchi SV, Miller CM, Fairweather SJ, Cai Y, Smith NC, et al. Cationic amino acid transporters play key roles in the survival and transmission of apicomplexan parasites. Nat Commun. 2017;8:14455; doi: 10.1038/ncomms14455.

8. Rajendran E, Clark M, Goulart C, Steinhöfel B, Tjhin ET, Gross S, et al. Substrate-mediated regulation of the arginine transporter of Toxoplasma gondii. PLoS Pathog. 2021;17 8:e1009816; doi: 10.1371/journal.ppat.1009816.

9. Fairweather SJ, Rajendran E, Blume M, Javed K, Steinhöfel B, McConville MJ, et al. Coordinated action of multiple transporters in the acquisition of essential cationic amino acids by the intracellular parasite Toxoplasma gondii. PLoS Pathog. 2021;17 8:e1009835; doi: 10.1371/journal.ppat.1009835.

10. De Koning HP, Al-Salabi MI, Cohen AM, Coombs GH, Wastling JM. Identification and characterisation of high affinity nucleoside and nucleobase transporters in Toxoplasma gondii. Int J Parasitol. 2003;33 8:821-31; doi: 10.1016/s0020-7519(03)00091-2.

11. Schwab JC, Afifi Afifi M, Pizzorno G, Handschumacher RE, Joiner KA. Toxoplasma gondii tachyzoites possess an unusual plasma membrane adenosine transporter. Molecular and biochemical parasitology. 1995;70 1-2:59-69; doi: 10.1016/0166-6851(95)00005-l.

12. Erler H, Ren B, Gupta N, Beitz E. The intracellular parasite Toxoplasma gondii harbors three druggable FNT-type formate and l-lactate transporters in the plasma membrane. J Biol Chem. 2018;293 45:17622-30; doi: 10.1074/jbc.RA118.003801.

13. Wan W, Dong H, Lai DH, Yang J, He K, Tang X, et al. The Toxoplasma micropore mediates endocytosis for selective nutrient salvage from host cell compartments. Nat Commun. 2023;14 1:977; doi: 10.1038/s41467-023-36571-4.

14. Lourido S, Moreno SN. The calcium signaling toolkit of the Apicomplexan parasites Toxoplasma gondii and Plasmodium spp. Cell Calcium. 2015;57 3:186-93; doi: 10.1016/j.ceca.2014.12.010.

15. Waller RF, Keeling PJ, Donald RG, Striepen B, Handman E, Lang-Unnasch N, et al. Nuclear-encoded proteins target to the plastid in Toxoplasma gondii and Plasmodium falciparum. Proc Natl Acad Sci U S A. 1998;95 21:12352-7; doi: 10.1073/pnas.95.21.12352.

16. Brooks CF, Johnsen H, van Dooren GG, Muthalagi M, Lin SS, Bohne W, et al. The toxoplasma apicoplast phosphate translocator links cytosolic and apicoplast metabolism and is essential for parasite survival. Cell Host Microbe. 2010;7 1:62-73; doi: 10.1016/j.chom.2009.12.002.

17. Chen P, Chen Y, Xia N, Fan B, Niu Z, He Z, et al. A pyruvate transporter in the apicoplast of apicomplexan parasites. Proceedings of the National Academy of Sciences of the United States of America. 2024;121 25:e2314314121; doi: 10.1073/pnas.2314314121.

18. Dong H, Yang J, He K, Zheng WB, Lai DH, Liu J, et al. The Toxoplasma monocarboxylate transporters are involved in the metabolism within the apicoplast and are linked to parasite survival. eLife. 2024;12; doi: 10.7554/eLife.88866.

19. Lyu C, Chen Y, Meng Y, Yang J, Ye S, Niu Z, et al. The Mitochondrial Pyruvate Carrier Coupling Glycolysis and the Tricarboxylic Acid Cycle Is Required for the Asexual Reproduction of Toxoplasma gondii. Microbiol Spectr. 2023;11 2:e0504322; doi: 10.1128/spectrum.05043-22.

20. Arnold CS, Alazzi AM, Shunmugam S, Janouškovec J, Berry L, Charital S, et al. A P5-ATPase, TgFLP12, diverging from plant chloroplast lipid transporters mediates apicoplast fatty export in Toxoplasma. Nature communications. 2025;16 1:5538; doi: 10.1038/s41467-025-61155-9.

21. Qian J, Zhao T, Guo L, Li S, He Z, He M, et al. Mitochondrial ADP/ATP Carrier 1 Is Important for the Growth of Toxoplasma Tachyzoites. Microbiology spectrum. 2023;11 3:e0004023; doi: 10.1128/spectrum.00040-23.

22. Shrivastava D, Abboud E, Ramchandra JP, Jha A, Marq JB, Chaurasia A, et al. ATM1, an essential conserved transporter in Apicomplexa, bridges mitochondrial and cytosolic [Fe-S] biogenesis. PLoS Pathog. 2024;20 9:e1012593; doi: 10.1371/journal.ppat.1012593.

23. Maclean AE, Sloan MA, Renaud EA, Argyle BE, Lewis WH, Ovciarikova J, et al. The Toxoplasma gondii mitochondrial transporter ABCB7L is essential for the biogenesis of cytosolic and nuclear iron-sulfur cluster proteins and cytosolic translation. mBio. 2024;15 10:e0087224; doi: 10.1128/mbio.00872-24.

24. Qin B, Fan B, Li Y, Wang Y, Shen B, Xia N. An endoplasmic reticulum localized acetyl-CoA transporter is required for efficient fatty acid synthesis in Toxoplasma gondii. Open Biol. 2024;14 11:240184; doi: 10.1098/rsob.240184.

25. Varadi M, Anyango S, Deshpande M, Nair S, Natassia C, Yordanova G, et al. AlphaFold Protein Structure Database: massively expanding the structural coverage of protein-sequence space with high-accuracy models. Nucleic acids research. 2022;50 D1:D439-d44; doi: 10.1093/nar/gkab1061.

26. Caffaro CE, Koshy AA, Liu L, Zeiner GM, Hirschberg CB, Boothroyd JC. A nucleotide sugar transporter involved in glycosylation of the Toxoplasma tissue cyst wall is required for efficient persistence of bradyzoites. PLoS Pathog. 2013;9 5:e1003331; doi: 10.1371/journal.ppat.1003331.

27. Bandini G, Leon DR, Hoppe CM, Zhang Y, Agop-Nersesian C, Shears MJ, et al. O-Fucosylation of thrombospondin-like repeats is required for processing of microneme protein 2 and for efficient host cell invasion by Toxoplasma gondii tachyzoites. J Biol Chem. 2019;294 6:1967-83; doi: 10.1074/jbc.RA118.005179.
